# Discovery and Biosynthesis of Bolagladins: Unusual Lipodepsipeptides from *Burkholderia gladioli* Clinical Isolates**

**DOI:** 10.1002/anie.202009110

**Published:** 2020-09-23

**Authors:** Yousef Dashti, Ioanna T. Nakou, Alex J. Mullins, Gordon Webster, Xinyun Jian, Eshwar Mahenthiralingam, Gregory L. Challis

**Affiliations:** ^1^ Department of Chemistry University of Warwick Coventry CV4 7AL UK; ^2^ Microbiomes, Microbes and Informatics Group Organisms and Environment Division School of Biosciences Cardiff University Cardiff CF103 AT UK; ^3^ Warwick Integrative Synthetic Biology Centre University of Warwick Coventry CV4 7AL UK; ^4^ Department of Biochemistry and Molecular Biology, ARC Centre of Excellence for Innovations in Peptide and Protein Science Monash University Clayton VIC 3800 Australia; ^5^ Current address: The Centre for Bacterial Cell Biology Biosciences Institute Medical School Newcastle University Newcastle upon Tyne NE2 4AX UK

**Keywords:** biosynthesis, fatty acids, genome mining, natural products, nonribosomal peptide synthetase

## Abstract

Two *Burkholderia gladioli* strains isolated from the lungs of cystic fibrosis patients were found to produce unusual lipodepsipeptides containing a unique citrate‐derived fatty acid and a rare dehydro‐β‐alanine residue. The gene cluster responsible for their biosynthesis was identified by bioinformatics and insertional mutagenesis. In‐frame deletions and enzyme activity assays were used to investigate the functions of several proteins encoded by the biosynthetic gene cluster, which was found in the genomes of about 45 % of *B. gladioli* isolates, suggesting that its metabolic products play an important role in the growth and/or survival of the species. The Chrome Azurol S assay indicated that these metabolites bind ferric iron, which suppresses their production when added to the growth medium. Moreover, a gene encoding a TonB‐dependent ferric‐siderophore receptor is adjacent to the biosynthetic genes, suggesting that these metabolites may function as siderophores in *B. gladioli*.

## Graphical abstract

Bolagladins A and B were discovered as products of a cryptic nonribosomal peptide synthetase in two *Burkholderia gladioli* strains isolated from the lungs of cystic fibrosis patients. These metabolites contain a unique citrate‐primed fatty acid and a rare dehydro‐β‐alanine residue. A combination of bioinformatics analysis and genetic and biochemical experiments illuminated the mechanisms for the installation of these unusual structural features.

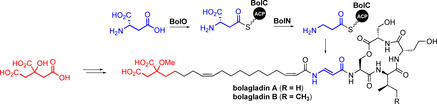

## Introduction

Iron is an essential element for most organisms. In biological systems, iron is mainly present in two oxidation states: ferrous and ferric.^[1]^ The interconversion of the ferrous and ferric oxidation states is essential for a variety of redox reactions and electron transfer processes in cells.^[2]^ However, despite the relatively high abundance of iron in the Earth's crust, it is not readily bioavailable. This is because it is predominantly in the ferric form, which has low aqueous solubility.^[3]^ Moreover, sequestration by proteins such as hemoglobin, ferritin, transferrin, and lactoferrin also contributes to low iron availability in mammals.^[3]^ As a consequence, microorganisms have evolved several strategies for obtaining iron from the environment and their hosts. One strategy involves the production of siderophores, specialised metabolites with a high affinity for ferric iron.^[4]^ In Gram‐negative bacteria, ferric‐siderophore complexes are typically transported into the periplasm by TonB‐dependent outer membrane receptors. Periplasmic binding proteins then shuttle the ferric‐siderophore complexes to the inner membrane, where ATP‐binding cassette (ABC) transporters transfer them into the cytoplasm.^[5]^ Once in the cytoplasm, iron is released from the ferric‐siderophore complex by reduction to the ferrous form and/or hydrolytic cleavage of the ligand.^[5a]^

Several investigations of pathogenic microorganisms have identified a strong link between siderophore production and virulence.^[6]^ In the early stages of infection, siderophores play a key role in sequestering iron from host tissues. The identification of siderophores produced by pathogenic microorganisms is therefore an important field of study because interference with siderophore‐mediated iron uptake is an attractive strategy for attenuating virulence.^[7]^ The discovery of novel siderophores is also relevant to other therapeutic applications. For example, desferrioxamine B (marketed as Desferal), a siderophore produced by numerous *Streptomyces* species,^[8]^ is used to treat iron overload resulting from multiple blood transfusions and aluminium toxicity due to dialysis. However, because Desferal is administered by injection and has multiple side effects, orally available alternatives with fewer side effects are actively being sought.^[9]^ In addition to clinical uses, siderophores show promise for environmental and biotechnological applications.^[10]^

**Figure 1:**
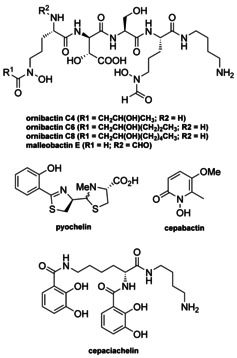
Structure of ornibactins, malleobactin E, pyochelin, cepaciachelin, and cepabactin identified from pathogenic strains of *Burkholderia*.

*Burkholderia* is a genus of highly diverse Gram‐negative bacteria. Species belonging to this genus have been isolated from soil, water, plants, animals and humans.^[11]^ Several investigations have shown that they are prolific producers of specialised metabolites,^[12]^ and genome sequence analyses have highlighted their underexplored biosynthetic potential.^[13]^ To date, pathogenic *Burkholderia* species have been reported to produce siderophores belonging to four distinct structural classes (ornibactins/malleobactins, pyochelin, cepabactin, and cepaciachelin; Figure 1).^[14]^ Members of the *cepacia* complex and *pseudomallei* group produce one or more of these siderophores. In contrast, *Burkholderia gladioli* does not produce any of these metabolites and was long believed to employ siderophore‐independent mechanisms for iron acquisition.^[15]^ However, Hertweck and co‐workers recently reported that three *B. gladioli* environmental isolates produce the diazeniumdiolate siderophore gladiobactin.^[16]^

We have recently discovered that *B. gladioli* BCC0238, isolated from the sputum of a child with cystic fibrosis (CF), produces several specialised metabolites. These include gladiolin, a novel macrolide with potent activity against drug resistant *Mycobacterium tuberculosis* clinical isolates,^[12i]^ and icosalide A1,^[17]^ an asymmetric lipopeptodiolide antibiotic originally isolated from an *Aureobasidium* species fungus,^[18]^ but subsequently shown to be produced by various strains of *B. gladioli* including one associated with the fungus.[^17^, ^19^] Analysis of the *B. gladioli* BCC0238 complete genome sequence identified several gene clusters encoding cryptic nonribosomal peptide synthetase (NRPS), polyketide synthase (PKS) and hybrid NRPS‐PKS assembly lines.^[12g]^ Here we report the discovery of bolagladins A and B, novel lipodepsipeptides containing a unique citrate‐derived fatty acid and a rare dehydro‐β‐alanine residue, as the metabolic products of one of these gene clusters. A combination of comparative bioinformatics analyses, targeted gene deletions, and enzyme activity assays elucidated several key steps in the biosynthesis of these unusual natural products, which we propose based on several lines of evidence may function as siderophores.

## Results and Discussion

### Isolation and Structure Elucidation of the Bolagladins

**Figure 2:**
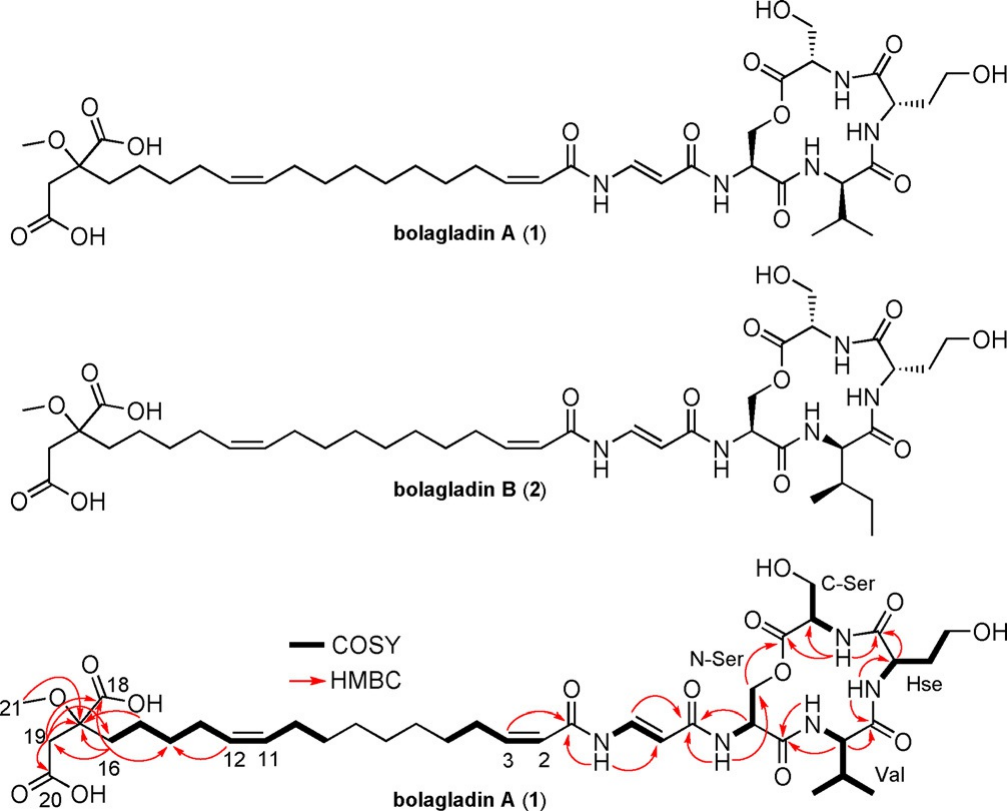
Structures of bolagladin A (**1**) and B (**2**). COSY and key HMBC correlations are shown for bolagladin A.

*B. gladioli* BCC0238 produces gladiolin and icosalide A1 when grown on a solid minimal medium containing glycerol as the sole carbon source.[^12i^, ^17^] Two novel metabolites were identified by UHPLC‐ESI‐Q‐TOF‐MS analysis of extracts from cultures of a gladiolin nonproducing mutant of *B. gladioli* BCC0238 (with an in‐frame deletion in the region of *gbnD1* encoding the ER domain) grown on BSM medium containing ribose as an additional carbon source alongside glycerol. The metabolites were isolated as amorphous solids from ethyl acetate extracts of 3 day cultures (1 L total volume) using semipreparative HPLC, and their planar structures were elucidated using a combination of HR‐ESI‐MS and 1D/2D NMR experiments (Figure 2; Tables S2 and S3, Figures S1–S14).

The molecular formulae of the two metabolites, which we named bolagladins A **1** and B **2**, due to their bolaamphiphilic nature, were established as C_39_H_61_N_5_O_14_ (calculated for C_39_H_62_N_5_O_14_^+^: 824.4288, found: 824.4284) and C_40_H_63_N_5_O_14_ (calculated for C_40_H_64_N_5_O_14_^+^: 838.4444, found: 838.4447), respectively, using positive ion ESI‐Q‐TOF MS (Figure S17). Four resonances with chemical shifts characteristic of protons attached to C2 in α‐amino acids (*δ*_H_ 4.17, 4.79, 5.36, and 5.52) correlated with signals attributable to exchangeable N−H protons (*δ*_H_ 8.17, 8.70, 9.91, and 9.99, respectively) in the COSY spectrum of bolagladin A **1**. Further analysis of ^1^H NMR and COSY spectra identified four α‐amino acid spin systems, which in conjunction with HSQC and HMBC data, were assigned to a valine residue, a homoserine (Hse) residue and two serine residues in the sequence Ser‐Hse‐Val‐Ser (Figure 2). A ^3^ *J*_CH_ correlation from the β‐protons of the N‐terminal Ser (N‐Ser) residue (*δ*_H_ 4.73 and 4.90) to the carbonyl carbon of the C‐terminal Ser (C‐Ser) residue (*δ*_C_ 170.0) established the presence of a tetradepsipeptide (Figure 2). Analysis of the COSY and HMBC data also revealed a dehydro‐β‐alanine (Dba) residue, with a coupling constant of 14.0 Hz between the α and β protons indicating that the double bond is *E*‐configured. The only other natural products reported to contain a Dba residue are the enamidonins isolated from *Streptomyces* species.^[20]^ ^2^ *J*_CH_ correlations were observed between the N−H proton of the N‐Ser residue in the tetradepsipeptide (*δ*_H_ 9.99) and the carbonyl carbon of the Dba residue (*δ*_C_ 168.3), and the N−H proton of Dba residue (*δ*_H_ 11.56) and the carbonyl carbon of a fatty acid residue (*δ*_C_ 165.1). This indicates that the Dba residue links the fatty acid residue to the tetradepsipeptide. The fatty acid residue is very unusual, with *Z*‐configured double bonds spanning C2–C3 and C11–C12 (based on CH=CH coupling constants of 11.5 and 13.5 Hz, respectively), and two carboxylic acid groups and a methoxy group appended to its tail (Figure 2).

The absolute configurations of the α‐amino acid residues in bolagladin A **1** were determined as l‐Ser‐d‐Val‐l‐Hse‐l‐Ser using Marfey's method^[21]^ (Figure S15). Comparison of the NMR spectroscopic data for bolagladins A **1** and B **2** showed that the Val residue in the former is substituted by an Ile residue in the latter, consistent with the difference of CH_2_ in the molecular formulae. The absolute configurations of the α‐amino acids in bolagladin B (**2**) were similarly shown to be l‐Ser‐d‐allo‐Ile‐l‐Hse‐l‐Ser by Marfey's method (Figure S15), using a C3 column instead of a C18 column to separate the Marfey's derivatives of d‐Ile and d‐allo‐Ile (Figure S15).^[22]^

Overall, the structures of bolagladins A **1** and B **2** are highly unusual, containing a unique fatty acid residue with several polar functional groups appended to its tail, linked via a rare Dba residue to a depsitetrapeptide. We thus sought to develop an understanding of bolagladin biosynthesis, focusing on the origin of the starter unit for the fatty acid assembly and the mechanism of Dba incorporation.

### Bolagladin Biosynthesis

**Figure 3:**
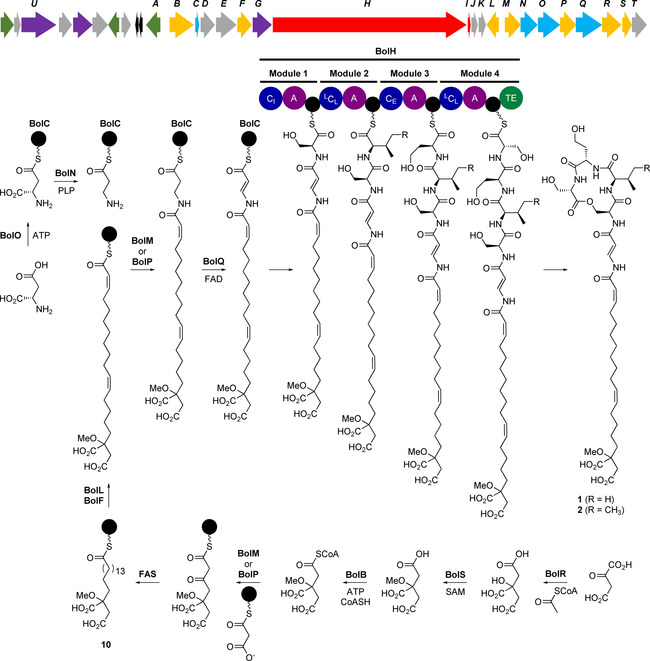
Organization of the bolagladin biosynthetic gene cluster (*bolA‐T*) and proposed pathway for bolagladin biosynthesis. The precise timing of *O*‐methylation of the citrate‐derived starter unit for fatty acid biosynthesis remains to be established, but it is postulated to be an early step. Abbreviations are as follows: A, adenylation domain; TE, thioesterase domain; C_I_, chain initiating condensation domain; ^L^C_L_, chain elongating condensation domain utilizing two l‐configured aminoacyl thioesters; C_E_, bifunctional epimerization–condensation domain; FAS, fatty acid synthase. Acyl carrier proteins (ACPs) and peptidyl carrier protein (PCP) domains are shown as black circles.

Bioinformatics analysis of the *B. gladioli* BCC0238 genome sequence identified the putative bolagladin (*bol*) biosynthetic gene cluster (Figure 3 and Table S4), which contains a gene (*bolH*) encoding a tetramodular NRPS. Sequence analysis of the adenylation (A) domains in this NRPS indicated that modules 1–4 activate l‐Ser, l‐Val/l‐Ile, l‐Hse and l‐Ser, respectively (Table S5),^[23]^ suggesting the NRPS assembles the depsipeptide portion of the bolagladins. Similarly, phylogenetic analysis of the BolH condensation (C) domains indicated that they belong to the following groups.^[24]^ Module 1: chain initiating (C_I_—links external acyl donor and l‐configured acyl acceptor); modules 2 and 4: chain elongating (^L^C_L_—links l‐configured acyl donor and acceptor); and module 3: bifunctional condensation/epimerization (C_E_— epimerizes acyl donor to d‐configuration and links with l‐configured acyl acceptor; Figure 3). This is consistent with the structure elucidation data, which indicate that the bolagladins contain l‐Ser, l‐Hse and d‐Val/d‐allo‐Ile.

**Figure 4:**
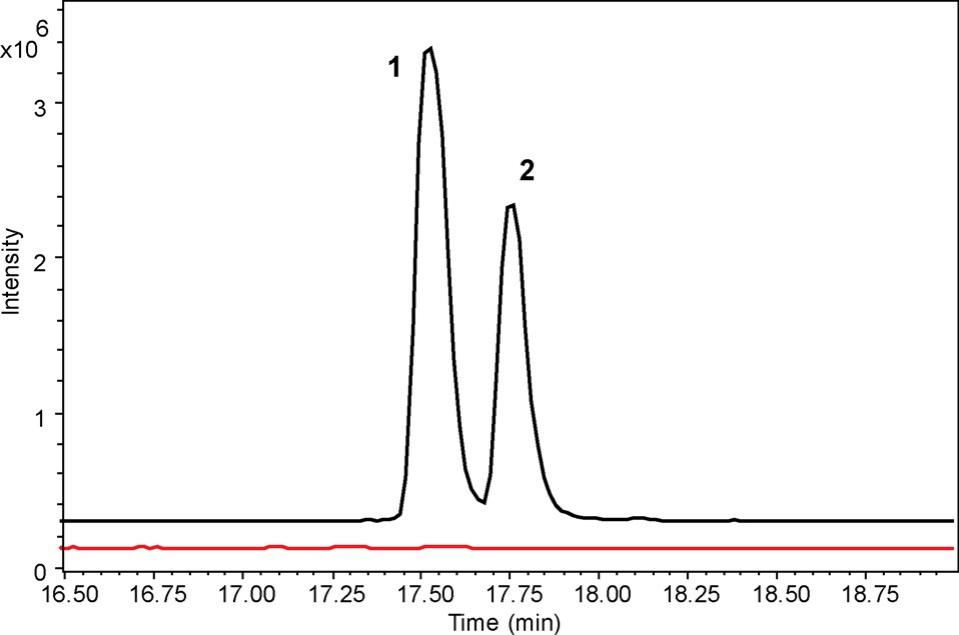
Comparison of the extracted ion chromatograms for *m*/*z* 824.4288 (**1**) and 838.4444 (**2**) from UHPLC‐ESI‐Q‐TOF MS analyses of culture extracts from *B. gladioli* BCC0238 *ΔgbnD1_ER* (black) and *B. gladioli* BCC0238 *ΔgbnD1_ER‐ΩbolH* (red). Insertional mutagenesis in *bolH* confirmed the involvement of the *bol* cluster in bolagladin production.

To verify the involvement of this gene cluster in bolagladin biosynthesis, we inactivated *bolH* in *B. gladioli* BCC0238 *ΔgbnD1_ER* via insertional mutagenesis. LC–MS analysis confirmed that the production of bolagladins A **1** and B **2** was abolished in the mutant (Figure 4).

**Figure 5:**
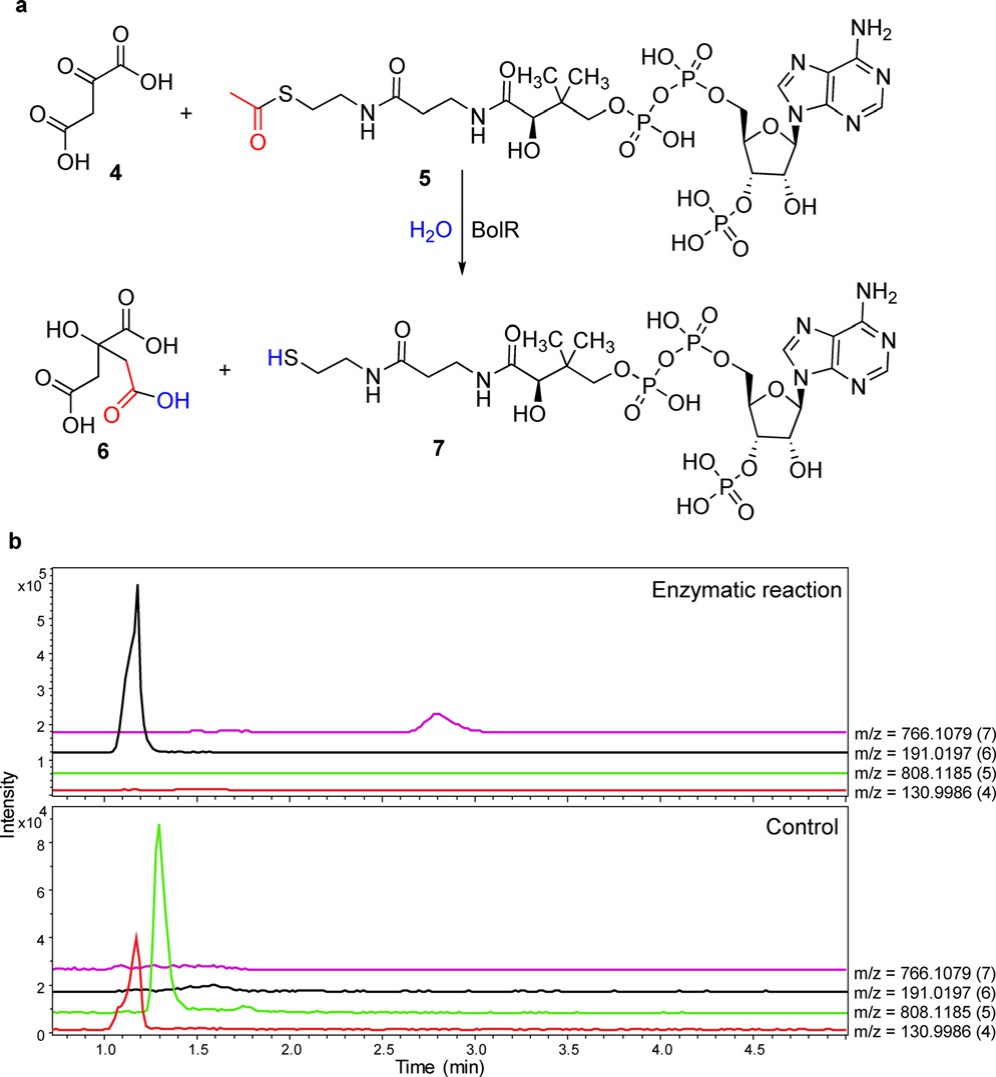
a) Reaction catalyzed by BolR. b) Extracted ion chromatograms for *m*/*z* 130.9986, 808.1185, 191.0197, and 766.1079 corresponding to [*M*−H]^−^ for **4**, **5**, **6**, and **7** from negative ion mode UHPLC‐ESI‐Q‐TOF MS analyses of the BolR‐catalyzed condensation of oxaloacetate with acetyl‐CoA (top) and a negative control reaction containing BolR inactivated by boiling for 15 min prior to addition (bottom). The measured *m*/*z* values for citrate and co‐enzyme A were 191.0195 and 766.1076, respectively.

Putative functions were assigned to the proteins encoded by the *bolA*‐*bolT* genes flanking *bolH* on the basis of sequence analyses (Table S4). This enabled us to propose a plausible pathway for bolagladin biosynthesis (Figure 3). To validate this pathway experimentally, we envisioned examining the effect of in‐frame deletions in key biosynthetic genes on bolagladin production. However, this proved to be challenging and time consuming in *B. gladioli* BCC0238 (see Supporting Information), so we screened other genome‐sequenced *B. gladioli* isolates containing the *bol* locus (see below) to establish whether they i) produce the bolagladins and ii) are amenable to construction of in‐frame deletions. *B. gladioli* BCC1622 (also isolated from a CF lung infection) was found to meet both criteria and an analogous gladiolin nonproducing mutant of this strain (*B. gladioli* BCC1622 *ΔgbnD1_ER*) was created to enable in‐depth functional studies of selected bolagladin biosynthetic genes. BolR shows sequence similarity to citrate synthase, a key enzyme in the Krebs cycle, which catalyzes the condensation of acetyl‐CoA with oxaloactetate and hydrolysis of the resulting thioester to form citrate (Figure 5 a).^[25]^ This suggests that citryl‐CoA may serve as the starter unit for assembly of the unusual fatty acid residue incorporated into the bolagladins (Figure 3). To verify the function of BolR, we overproduced it in *E. coli* as an N‐terminal His_6_‐fusion and purified it to homogeneity (see Supporting Information). Incubation of the purified protein with oxaloacetate (**4**) and acetyl‐CoA (**5**) for 2 h at room temperature resulted in complete conversion to products with molecular formulae corresponding to citric acid (**6**) and coenzyme A **7** (Figure 5 b).

**Figure 6:**
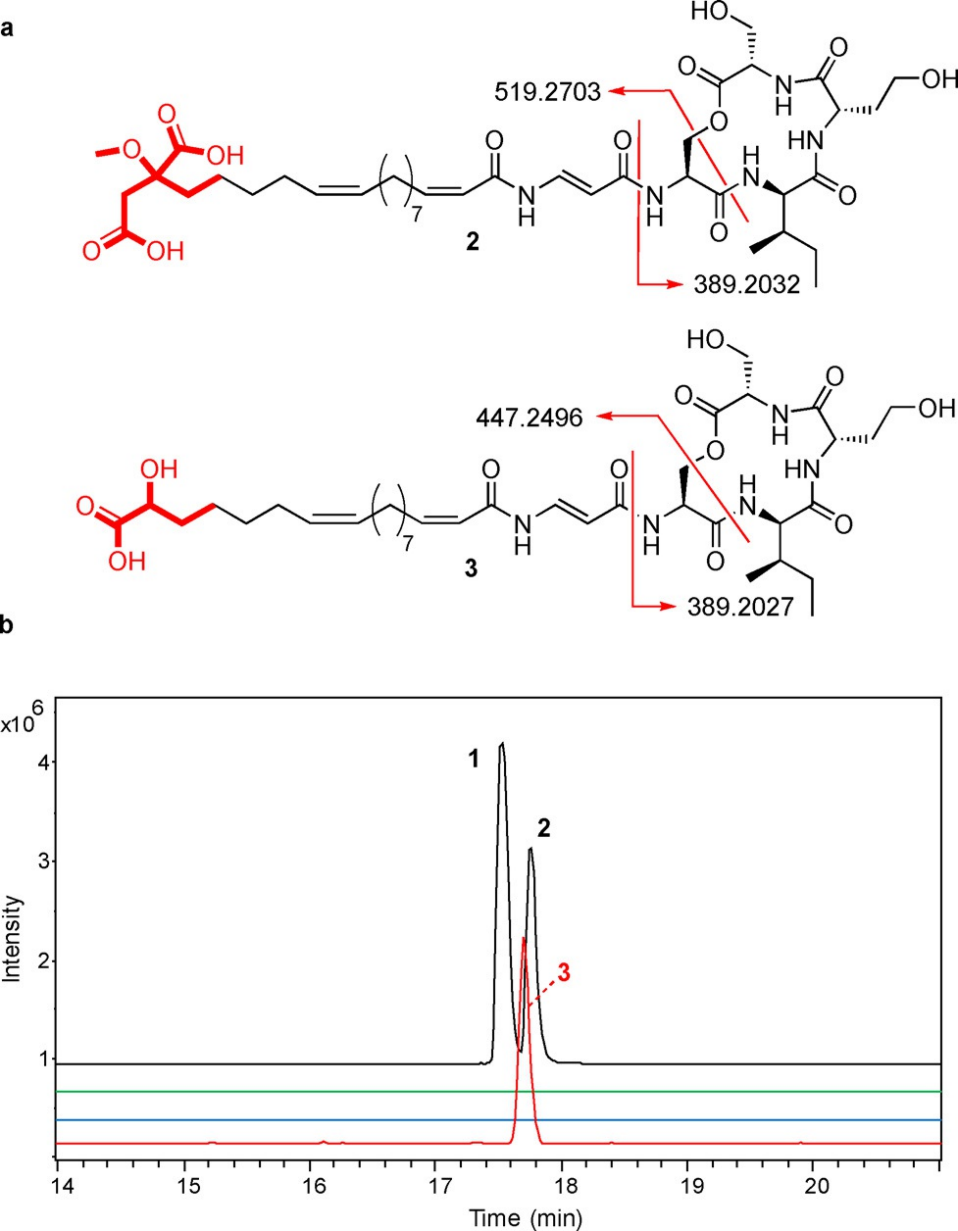
a) Comparison of fragment ions observed for metabolite **3** and bolagladin B (**2**) in MS/MS analyses. b) Extracted ion chromatograms (EICs) from UHPLC‐ESI‐Q‐TOF MS analyses of extracts from *B. gladioli* BCC1622 *ΔgbnD1_ER* and a *bolR* deletion mutant. From top to bottom, these are: EIC at *m*/*z* 824.4288 (corresponding to [*M*+H]^+^ for **1**) and 838.4444 (corresponding to [*M*+H]^+^ for **2**) for the wild‐type strain; EIC at *m*/*z* 824.4288 and 838.4444 for the mutant; EIC at *m*/*z* 766.4233 (corresponding to [*M*+H]^+^ for **3**) for the wild‐type strain; and EIC at *m*/*z* 766.4233 for the mutant.

To probe the role played by citrate in bolagladin biosynthesis, we constructed an in‐frame deletion in *bolR*. The resulting mutant was unable to assemble bolagladins A and B, but produced a new metabolite with the molecular formula C_37_H_59_N_5_O_12_ (calculated for C_37_H_60_N_5_O_12_^+^: 766.4233, found: 766.4238; Figures 6 and S16). The production level of this metabolite was insufficient for NMR spectroscopic analysis. We therefore conducted LC–MS/MS analyses, which indicated the metabolite has the same depsipeptide core as bolagladin B **2** (molecular formula C_40_H_63_N_5_O_14_; Figures 6 and S16) and that the structural differences must lie in the fatty acid and/or Dba residues. However, because the new metabolite contains three fewer carbon atoms, four fewer hydrogen atoms and two fewer oxygen atoms than bolagladin B, but the same number of nitrogen atoms, these differences cannot be due to modification or loss of the Dba residue. The most plausible structure for this metabolite is **3**, in which the fatty acid residue is derived from an oxaloacetyl‐CoA starter unit that is subsequently reduced (Figure 6). This suggests that BolR may catalyze the condensation of acetyl‐CoA with the keto group of oxaloacetyl‐CoA in bolagadin biosynthesis (Figure S17), but further experiments will be required to confirm this.

**Figure 7:**
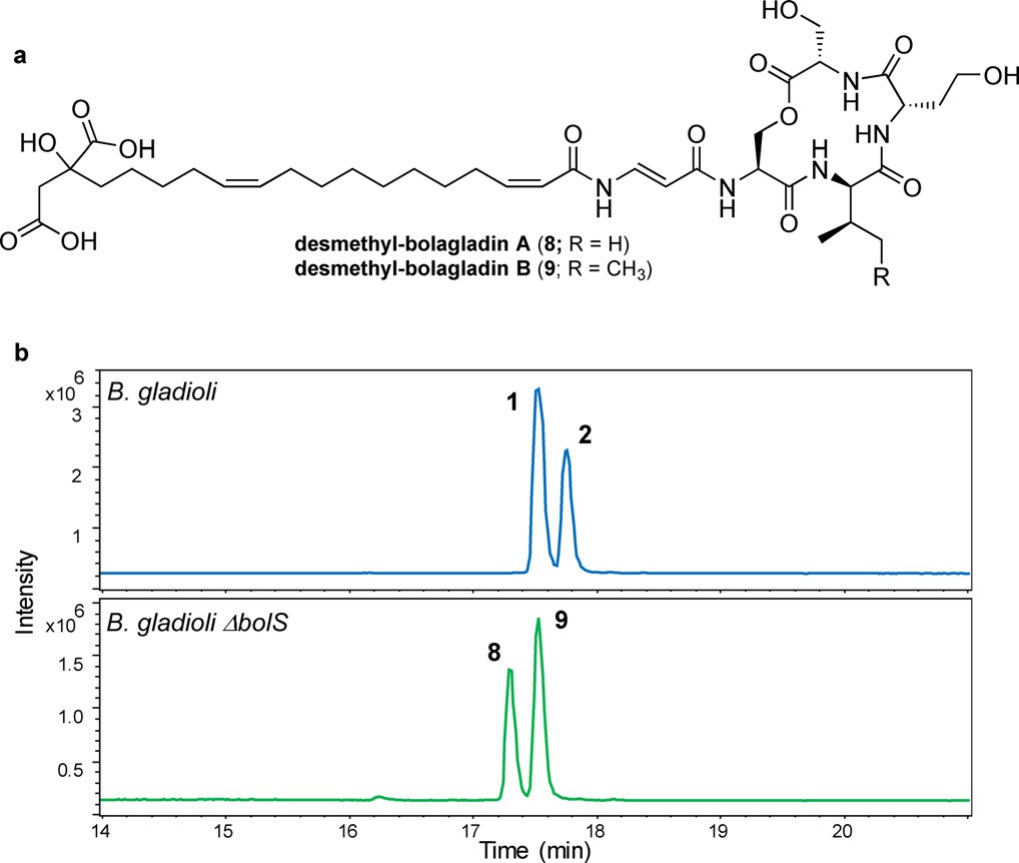
a) Structures of desmethyl‐bolagladins A **8** and B **9** accumulated in the *bolS* mutant of *B. gladioli* BCC1622 *ΔgbnD1_ER*. b) Top: Extracted ion chromatogram at *m*/*z* 824.4288 (corresponding to [*M*+H]^+^ for **1**) and 838.4444 (corresponding to [*M*+H]^+^ for **2**) from UHPLC‐ESI‐Q‐TOF MS analyses of culture extracts from *B. gladioli* BCC1622 *ΔgbnD1_ER*. Bottom: Extracted ion chromatogram at *m*/*z* 810.4131 (corresponding to [*M*+H]^+^ for **8**) and 824.4288 (corresponding to [*M*+H]^+^ for **9**) from UHPLC‐ESI‐Q‐TOF MS analyses of culture extracts from the *bolS* mutant of *B. gladioli* BCC1622 *ΔgbnD1_ER*.

We postulated that BolS, which shows sequence similarity to *S*‐adenosyl‐l‐methione (SAM) dependent methyltransferases, catalyzes methylation of the hydroxyl group in the citrate‐derived starter unit at some point during bolagladin biosynthesis. To test this hypothesis, we constructed an in‐frame deletion in *bolS*. The production of bolagladins A **1** and B **2** was abrogated in the resulting mutant and two new metabolites with the molecular formulae C_38_H_59_N_5_O_14_ (calculated for C_38_H_60_N_5_O_14_^+^: 810.4131, found: 810.4124) and C_39_H_61_N_5_O_14_ (calculated for C_39_H_62_N_5_O_14_^+^: 824.4288, found: 824.4296) were produced (Figures 7 and S16). LC–MS/MS analyses indicated that these compounds are congeners of **1** and **2**, lacking a CH_2_ group from the fatty acid or Dba residues (Figure S17). Although production levels of these metabolites were low, we were able to purify a sufficient quantity of the bolagladin B congener for NMR spectroscopic analysis (Table S6, Figures S18–S23). Differences were observed in the ^13^C chemical shifts of the resonances due to C16, C17, C18, C19 and C20 in the fatty acid residue and the signals attributed to the *O*‐methyl group in bolagladin B were absent from the ^1^H and ^13^C spectra. We therefore conclude that the two metabolites accumulated in the *bolS* mutant are desmethyl‐bolagladins A **8** and B **9**. The low levels of **8** and **9** produced by the mutant suggest *O*‐methylation of the citrate‐derived starter unit is an early step in bolagladin biosynthesis, rather than a late‐stage modification. However, the precise timing of this reaction remains to be determined.

Citrate (or its *O*‐methylated derivative) is proposed to be converted to the corresponding coenzyme A thioester by BolB, which shows similarity to acyl‐CoA synthetases (Figure 3 and Table S4). Either BolM or BolP, both of which show similarity to β‐ketoacyl synthase III (KAS III) enzymes that typically initiate fatty acid biosynthesis in bacteria (Table S4),^[26]^ could then catalyse the elongation of citryl‐CoA (or its O‐methylated derivative) with a malonyl group attached to the primary metabolic fatty acid synthase (FAS) acyl carrier protein (ACP) (Figure 3). Interestingly, the conserved active site Cys residue is mutated to Ser in BolM and Thr in BolP. The functional significance of this is unclear, but an analogous Cys to Ser mutation is observed in DpsC, a KAS III homologue that has been reported to initiate assembly of the daunorubicin polyketide chain using a propionyl‐CoA starter unit.^[27]^ Further processing of the β‐ketothioester resulting from elongation of (*O*‐methyl)‐citryl‐CoA with malonyl‐ACP by the primary metabolic FAS would afford the saturated *O*‐methyl‐citrate‐primed fatty acyl‐ACP thioester **10** (Figure 3). The 11, 12 and 2, 3 double bonds are likely introduced into this thioester by BolF and BolL, which are similar to membrane‐associated fatty acid desaturases and acyl‐ACP desaturases, respectively (Figure 3 and Table S4), completing the assembly of the unusual fatty acid residue incorporated into the bolagladins.

The Dba residue of the bolagladins is postulated to derive from l‐aspartate, which we propose undergoes adenylation of its β‐carboxyl group catalyzed by BolO, followed by transfer onto BolC, a putative freestanding ACP (Figure 3). BolO shows similarity to VinN,^[28]^ which catalyzes adenylation of the β‐carboxyl group of β‐methyl‐aspartate and subsequent transfer to the freestanding ACP VinL in the biosynthesis of vicenistatin. Moreover, the Asp230 and Ser299/Arg331 residues in VinN, which are proposed to play a key role in recognition of the α‐amino and α‐carboxyl groups of β‐methyl‐aspartate, respectively, are conserved in BolO (Figure S24). In vicenistatin biosynthesis, the pyridoxal phosphate (PLP)‐dependent enzyme VinO catalyses decarboxylation of the β‐methyl‐isoaspartyl‐VinL thioester to form the corresponding α‐methyl‐β‐alanyl thioester.^[24]^ We propose that BolN, which shows sequence similarity to BtrK, a PLP‐dependent enzyme that catalyzes decarboxylation of an *iso*‐glutamyl‐ACP thioester in butirosin biosynthesis, plays an analogous role in bolagladin biosynthesis (Figure 3). In‐frame deletion of *bolN* abolished bolagladin production and it could not be restored by feeding β‐alanine to the mutant. This is consistent with the proposed roles of BolO, BolC and BolN and rules out β‐alanine as an intermediate in the biosynthetic pathway. The amino group of the β‐alanyl‐BolC thioester is proposed to be condensed with the citrate‐primed fatty acyl thioester by one of the KAS III homologues BolM or BolP (Figure 3).

**Figure 8:**
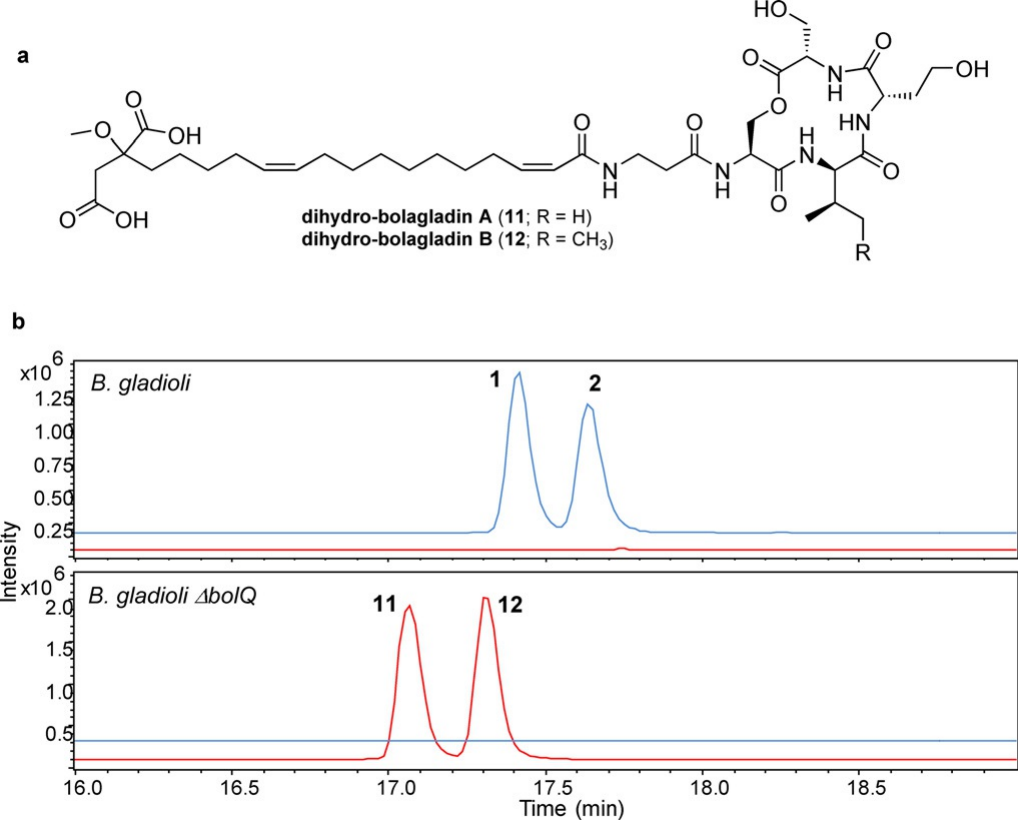
a) Structures of dihydro‐bolagladins A (**11**) and B (**12**) produced by the *bolQ* mutant of *B. gladioli* BCC1622 *ΔgbnD1_ER*. b) Extracted ion chromatograms at *m*/*z* 824.4288 and 838.4444 (in blue; corresponding to [*M*+H]^+^ for bolagladins A (**1**) and B (**2**), respectively), and 826.4444 and 840.4601 (in red, corresponding to [*M*+H]^+^ for dihydro‐bolagladins A (**11**) and B (**12**), respectively) from UHPLC‐ESI‐Q‐TOF MS analyses of culture extracts from *B. gladioli* BCC1622 *ΔgbnD1 _ER* and the *bolQ* mutant.

The sequence similarity of BolQ to acyl‐CoA dehydrogenases suggested it might be responsible for formation of the Dba residue via desaturation of the *N*‐acyl‐β‐alanyl‐BolC thioester. To test this hypothesis, we created an in‐frame deletion in *bolQ*, which abolished the production of bolagladins A and B and led to the accumulation of two new metabolites with the molecular formulae C_39_H_63_N_5_O_14_ and C_40_H_65_N_5_O_14_ (Figure 8). Purification and NMR spectroscopic analysis showed these metabolites are dihydro‐bolagladins A **11** and B **12**, in which the Dba residue has been replaced by β‐alanine (Tables S7 and S8, and Figures S25–S35). This is consistent with the proposed function of BolQ as a *N*‐acyl‐β‐alanyl thioester desaturase, although on the basis of these data the possibility of BolQ converting the β‐alanine residue to Dba at a later stage in bolagladin biosynthesis cannot be ruled out.

### The *bol* Locus is Widely Conserved in *B. gladioli*

**Figure 9:**
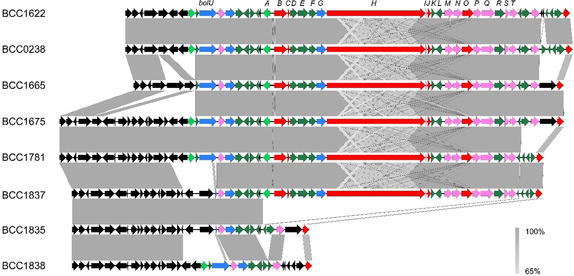
Conservation of the bolagladin biosynthetic gene cluster in *B. gladioli*. The gene cluster was extracted from six *B. gladioli* genome assemblies (BCC1622, BCC0238, BCC1665, BCC1675, BCC1781, and BCC1837, all of which are CF lung infection isolates) that highlight the diversity of gene insertions, deletions, and substitutions flanking the cluster. *B. gladioli* BCC1835 and BCC1838 were included as examples of strains lacking the bolagladin biosynthetic gene cluster. No variation in the *bolA*‐*bolT* genes was observed for strains containing the bolagladin biosynthetic gene cluster.

A local nucleotide BLAST search of 1318 genomes representing *Burkholderia*, *Paraburkholderia* and *Caballeronia* species showed *B. gladioli* is the only species containing the bolagladin biosynthetic gene cluster (as indicated by the presence of *bolH*).^[29]^ Read mapping of paired‐end Illumina reads from 234 *B. gladioli* strains against the *bol* gene cluster revealed that it is present in 105 strains. However, there is evidence of gene substitutions and deletions in the regions flanking the cluster (Figure 9). The *bolU* gene, encoding a putative TonB‐dependent ferric‐siderophore outer membrane receptor, is absent from five of the 105 *B. gladioli* genomes (BCC1837, BCC1843, BCC1861, BCC1870 and BCC1871) containing the bolagladin biosynthetic gene cluster and is present in 52 % (67 of 129) of genomes lacking it.

### Biological Function of the Bolagladins

Bolagladins A (**1**) and B (**2**) showed no activity at a concentration of 64 μg mL^−1^ in disc diffusion assays against any of the ESKAPE panel of bacterial pathogens,^[30]^ *Mycobacterium bovis* BCG, or *Candida albicans*. The 3‐methoxy‐1,4‐dicarboxylate moiety of the bolagladins is reminiscent of ferric‐iron‐chelating citrate residues in several siderophores, such as achromobactin, vibrioferrin, and staphyloferrin B.^[31]^ Moreover, *bolU* flanking the left end of the bolagladin biosynthetic gene cluster encodes a putative TonB‐dependent ferric‐siderophore outer membrane receptor. We thus hypothesised that the bolagladins could function as siderophores. Although ferric complexes of the bolagladins were too labile to observe in ESI‐Q‐TOF MS analyses, the chrome azurol S (CAS) assay indicated they are able to sequester ferric iron, albeit at higher concentrations than typical trishydroxamate siderophores (Figure S36).^[4]^

Siderophore production in microbes is usually regulated by iron availability. In iron‐deficient media, production is upregulated, whereas in iron replete media it is suppressed. Accordingly, the level of bolagladin production by *B. gladioli* BCC1622 *ΔgbnD1_ER* decreased as increasing concentrations of ferric iron were added to the medium (Figure S36). Combined with the results of the CAS assay, these data indicate that the bolagladins may function as siderophores in *B. gladioli*.

Siderophores often contribute to virulence in pathogenic bacteria. Thus, we used a *Galleria mellonella* wax moth infection model to investigate whether the bolagladins are a virulence factor in *B. gladioli*. Ornibactin was shown to contribute to virulence in *Burkholderia cenocepacia* using this and other models.[^15^, ^32^] However, no attenuation of virulence towards the wax moth larvae was observed in the *ΔbolN* mutant of *B. gladioli* BCC1622 *ΔgbnD1_ER*, relative to the parental strain (Figure S37).

A similar observation was reported for pyochelin deficient mutants of *B. cenocepacia* H111,^[32]^ leading to the hypothesis that some *Burkholderia* siderophores may play a role in metal homeostasis rather than virulence.^[33]^ To investigate this hypothesis, *B. cenocepacia* H111 pyochelin and ornibactin nonproducing mutants were grown alongside the wild‐type strain in media containing various metal salts (10–50 μM). The metal salts did not affect the wild‐type strain, but were toxic to the siderophore nonproducing mutants, indicating that pyochelin and ornibactin protect *B. cenocepacia* against metal ion toxicity.^[33]^ This finding was further supported by supplementing the medium with pyochelin and ornibactin, which reversed toxicity in the siderophore nonproducing mutants.^[33]^

We thus compared the tolerance of the *B. gladioli* BCC1622 *ΔgbnD1_ER* and *ΔgbnD1_ER‐ΔbolN* strains to salts of aluminium, zinc, cobalt, copper, cadmium, nickel and lead at various concentrations up to 100 μM. No decrease in metal ion tolerance was observed for the bolagladin nonproducing mutant relative to parent strain and the results of these experiments indicated that *B. gladioli* is much less susceptible to metal ion toxicity than *B. cenocepacia*. Further experiments demonstrated that *B. gladioli* could readily grow in media containing 1 mM Ni^2+^, Al^2+^ and Zn^2+^. Additional work is required to establish the genetic basis for this high degree of metal ion tolerance.

## Conclusion

Bacteria belonging to the *Burkholderia* genus are increasingly being recognised as an underexplored source of novel natural products.^[34]^ Using a combination of carbon source modification and inactivation of the biosynthetic pathway for gladiolin, which is produced at high titre and interferes with the detection of lower abundance metabolites, we have identified bolagladins A (**1**) and (**2**) as the metabolic product of a cryptic nonribosomal peptide biosynthetic gene cluster in *B. gladioli*. Similar approaches may prove useful for identifying the products of other cryptic *Burkholderia* biosynthetic gene clusters.

The bolagladins have very unusual structures, consisting of a unique fatty acid residue with several polar functional groups appended to its tail, linked via a very rare Dba residue to a depsitetrapeptide. Identification of the bolagladin biosynthetic gene cluster in *B. gladioli* BCC0238 and BCC1622 enabled us to probe the origin of these unusual structural features using a combination of detailed bioinformatics analysis, gene deletions and enzyme activity assays. Based on these studies, we propose that the fatty acid residue originates from a citryl‐CoA starter unit. To our knowledge, there is no precedent for the utilisation of citryl‐CoA as a starter unit in fatty acid or polyketide biosynthesis. We also hypothesise that the Dba residue derives from loading of aspartate onto a freestanding ACP. The resulting isoaspartyl thioester is proposed to undergo decarboxylation and N‐acylation, prior to being desaturated by a flavin‐dependent dehydrogenase, resulting in an *N*‐acyl‐Dba thioester starter unit for the NRPS that assembles the depsitetrapeptide. While loading of aspartate/β‐methyl‐aspartate onto a standalone ACP, followed by decarboxylation and N‐acylation, is a well‐established mechanism for provision of β‐aminoacyl starter units to type I modular PKSs,^[35]^ to our knowledge there is no precedent for this mechanism being employed to provide such starter units to NRPSs. Thus, our findings expand the scope of β‐aminoacyl starter unit biosynthetic machinery, which may prove useful in bioengineering approaches to natural product diversification.

The bolagladin biosynthetic gene cluster is present in approximately 45 % of *B. gladioli* genomes, but was not found in other *Burkholderia* species. This suggests the bolagladins play an important role in the adaption of *B. gladioli* to its environmental niches. Several lines of evidence indicate that the bolagladins may function as siderophores, further challenging the assumption that *B. gladioli* employs siderophore‐independent mechanisms for iron acquisition.[^15^, ^16^] While the mode of ferric iron binding to the bolagladins remains to be established, it seems likely that the two citrate‐derived carboxy and methoxy groups in the unusual fatty acid residue, and one or both of the side‐chain hydroxyl groups in the depsipetide are involved. Siderophores are known to play roles in virulence and metal ion tolerance in other *Burkholderia* species, but a *Galleria mellonella* infection model did not provide evidence that the bolagladins contribute to virulence in *B. gladioli* and bolagladin‐deficient mutants did not show reduced tolerance towards metal ions. Further studies are therefore required to develop a better understanding of the adaptive benefit the bolagladins confer on *B. gladioli*.

## Conflict of interest

The authors declare no conflict of interest.

## Supporting information

As a service to our authors and readers, this journal provides supporting information supplied by the authors. Such materials are peer reviewed and may be re‐organized for online delivery, but are not copy‐edited or typeset. Technical support issues arising from supporting information (other than missing files) should be addressed to the authors.

Supplementary
